# Genetic analysis of stay green related traits in maize with major gene plus polygenes mixed model

**DOI:** 10.1371/journal.pone.0303602

**Published:** 2024-10-03

**Authors:** Ran Zheng, Yuchen Zhou, Dan Lv, Bo Tong, Hongbing Luo

**Affiliations:** 1 College of Agronomy, Hunan Agricultural University, Changsha, Hunan, China; 2 Maize Engineering Technology Research Center of Hunan Province, Changsha, Hunan, China; KGUT: Graduate University of Advanced Technology, ISLAMIC REPUBLIC OF IRAN

## Abstract

Maize is one of the main food crops in the world, and cultivating high-yield and high-quality maize varieties is of great significance in addressing food security issues. Leaves are crucial photosynthetic organs in maize, and leaf senescence can result in the degradation of chlorophyll. This, in turn, impacts photosynthetic activity and the accumulation of photosynthetic products. Delaying leaf senescence and increasing carbon assimilation can enhance grain yield and biomass production. The stay green of maize is an important trait closely related to yield, feed quality and resistance. Therefore, this study employed multi-generation joint analysis of major genes and a polygene model to investigate the genetic inheritance of stay green-related traits. Four populations (P_1_, P_2_, F_1_ and F_2_) were obtained by crossing T01 (stay green) × Xin3 (non-stay green) and T01 (stay green) × Mo17 (non-stay green) under two environments. Six stay green-related traits, including visual stay green (VSG), number of green leaves (GLNM), SPAD value of ear leaf at anthesis (SPADS), SPAD value of ear leaf at maturity (SPADM), absolute green leaf area (GLAD), grain yield per plant (GYP), displayed continuous variations with kurtosis and skewness values of absolute value less than 1 and distribution close to normal. They were characterized by typical inheritance of quantitative traits, with these traits demonstrating the transgressive segregation. The correlation analysis among the traits revealed that five stay green traits have a positive impact on yield. VSG, GLNM and SPADM in the two populations were regulated by the two major genes of additive effects plus additive-dominance polygene model with a major gene heritability varying from 89.03 to 95.95% in the F_2_ generation. GLAD in TMF_2_ was controlled by two major genes of equal-additive dominance effects with high heritability (93.47%). However, in TXF_2_, GLAD was regulated by two major genes of additive-dominance interaction effects plus additive-dominance polygene model. These results provide important genetic information for breeding, which could guide the improvement of stay green-related traits. They also lay a foundation for quantitative trait loci mapping of the stay stay-green traits in maize.

## Introduction

Stay green is a phenomenon in plants where the leaves remain green in the late stage of plant growth and development due to delayed yellowing or senescence. In maize, this phenomenon was first reported by Willman et al. [1], where the maize leaves experienced delayed senescence, maintaining a vigorous photosynthesis rate at the initiation of the mature stage. The main evaluation indicators of leaf stay green phenotype include visual stay green (VSG: the proportion of green leaf area to the maximum leaf area at physiological maturity), absolute green leaf area duration (GLAD), relative green leaf area duration (GLAD), the number of green leaves (GLNM) and the chlorophyll content at physiological maturity [2–4]. Some stay-green plants experience delayed senescence, maintain the integrity of chloroplasts in the leaves, maintain normal photosynthesis rate, extended photosynthetic duration, improved photosynthetic efficiency, and increased yields due to the relatively complete system of active oxygen scavenging, osmotic regulation and photochemical activity at the late growth stage. Bekavac et al. [5] revealed a significant correlation between the green retention in maize and the length of vegetative growth, grain and leaf water content. Besides, the later the senescence of maize leaves, the slower the senescence rate, and the more beneficial it is to increase the grain weight and number of grains per ear [6, 7]. In addition, plant N levels are closely related to photosynthesis and leaf ageing because they are an essential component of chlorophyll [8]. The stay-green cultivar retained higher levels of reduced nitrogen and chlorophyll than a non-stay-green cultivar, which helped to accumulate more photosynthetic products during the grain-filling stage [9]. Under the conditions of N-deficiency stress, in particular, the stay-green grain’s capacity to increase yield was more obvious [10].

The combination of quantitative genetics and plant breeding and the application of mathematical statistics and genetic models to analyze the genetic laws of quantitative population traits are of great significance in providing the scientific basis for modern plant breeding [11–13]. The "major gene polygene genetic model separation and analysis method" has been established to identify the major genes, polygene effects, gene interactions [14], epistasis, and interactions between genes and environment [15, 16] in a quantitative trait genetic system. The major gene and polygene hybrid genetic model of quantitative traits in maize has mainly focused on yield, quality and stress resistance [17–22]. The genetic model has also been applied to study maize stay green-related traits, focusing mainly on chlorophyll content. Specifically, the chlorophyll content is controlled by the additive-dominance-epistatic polygene mixed genetic model [23]. In contrast, another report revealed that the chlorophyll content is controlled by the main gene polygene mixed genetic model [24]. Besides, the genetic laws obtained by the researchers vary depending on the study materials. The existing studies have mainly focused on the statistical genetic analysis of the genetic characteristics of a single character in a single environment. Until now, few reports have been on quantitative genetic analysis of multiple stay green traits in maize under multiple environments. In this study, the T01 maize inbred line (common parent) was crossed with Mo17 and Xin3 maize inbred lines, generating two F_2_ populations (TMF_2_ and TXF_2_) for phenotypic identification analysis in two environments (China, Chang sha, 2021, 2022). The genetic model and the mode of genetic action of the stay green trait in two maize inbred lines were identified using the main gene+polygene mixed genetic model analysis method. The findings in this study provide a basis for QTL location and trait improvement of the stay green-related trait.

## Materials and methods

### Plant materials

The stay-green maize P_1_ (maize inbred line T01) was used as the common male parent and crossed with two non-stay-green maize P_2_ (maize inbred lines Mo17 and Xin3). The obtained F_1_ generation was self-crossed to generate two F_2_ populations, TMF_2_ and TXF_2_. The T01, Mo17 and Xin3 seeds were provided by the Maize Engineering Technology Research Center of Hunan Province (China). The P_1_, P_2_, F_1_ and F_2_ generations were planted in the Dahu base in Liuyang, Hunan, China (28.2°N, 113.6°E) in April to July 2021 (E1) and 2022 (E2), respectively. 200 plants will be planted in each of these two populations in E1, and 400 plants will be planted in each of these two populations in E2. Using manual sowing method, the sowing depth is about 3.00 cm. The experimental site is located in a subtropical monsoon climate zone, with soil organic matter of 21.44 g/kg, available phosphorus of 118.25 mg/kg, total phosphorus of 1.45 g/kg, total nitrogen of 1.00 g/kg, available potassium of 120.00 mg/kg, and total potassium of 18.80 g/kg at 0–20 cm before sowing.

### Field measurement

Five stay green related traits (VSG, GLNM, SPADS, SPADM, and GLAD) were measured as previously described by Wang [4] and Van [25]. VSG (%): The ratio of green leaf area at maturity to green leaf area at flowering stage, GLNM: Number of green leaves per plant at maturity, SPADS and SPADM: Using the SPAD-502P instrument to measure the SPAD values during the flowering and maturity stages, respectively, GLAD (cm^2^): According to the field investigation, the number and area of green leaves per plant of maize at different dates after flowering were determined. Using the logistic equation (y = a/(1+e) ^ (b+cx)) to simulate the trend of single plant leaf area over time, and then calculates the cumulative value of green leaf area within 40 days after flowering, which is the definite integral value of the curve in the interval (0, 40), finally, calculates the daily average green leaf area accumulation as GLAD. In addition, GYP (g) was determined as the grain weight of mature maize.

### Statistical analysis

Phenotypic data were analyzed with Excel 2020 and SPSS 20.0. Multiple comparisons were conducted using the Duncan test, and significant differences were marked with different letters. The frequency distribution histograms of stay green related traits and grain yield per plant were generated by origin, using the R 4.2.0 statistical software performance. The correlation between five stay-green-related traits and GYP was analyzed using the multi-generation joint analysis method in the R software package SEA-G4F2 (https://cran.r-project.org/web/packages/SEA/index.html) and visualized using the analytics installation package. At the same time, the major gene+polygene hybrid genetic model was used to analyze five stay-green-related traits and GYP of parents, F_1_ and F_2_ populations. Subsequently, the maximum likelihood function value was calculated using the Likelihood method and converted to Akaike’s Information Criterion (AIC) value. Next, models with the lowest AIC value were selected as the candidate genetic models, the suitability of the sample distribution and the theoretical distribution of the model were tested. Based on the test results, the model with the least statistics reaching the significance level was selected as the most appropriate genetic model for the traits. Finally, the genetic parameters of the least squares method were used to analyze the test results.

## Results

### Descriptive statistics of stay-green-related traits and grain yield per plant

The stay green related traits, including VSG, GLNM, SPADS, SPADM, GLAD and GYP, were significantly different (*P* < 0.05) between the T01 and Mo17 maize inbred populations under the different environments. Precisely, the average values for the six traits were higher in T01 than in Mo17 (Table 1). There are varying degrees of bidirectional transgressive segregation phenomena in the six traits of TMF_2_ population. The coefficient of variation (CV) reflects the extent of variation between the F_2_ populations. In addition, the CV of SPADM was the highest (55.81%), followed by GYP, GLNM, VSG, GLAD, and lowest in SPADS (12.14%). Moreover, the absolute values of skewness and kurtosis of all traits in the TMF_2_ population in both environments were less than 1 except for GLNM under the E1 environment, implying a nearly normal distribution. GLNM also had a continuous multi-peak distribution, consistent with the major gene plus polygene characteristics, implying that there may be major genes.

**Table 1:** Descriptive analysis of stay-green-related traits and grain yield per plant in TMF_2_ and TXF_2_ population.

Population.	Trait	Environment	Parents			F_2_ population			
			T01	Mo17	Xin3	Mean±SD	Variable coefficient (%)	Skewness	Kurtosis
TMF_2_	VSG (%)	E1	59.40±4.57a	9.52±16.50b	-	51.39±25.66	49.93	0.05	-0.88
		E2	69.26±8.91a	6.06±5.25b	-	46.82±25.86	55.24	-0.23	-0.71
	GLNM	E1	6.33±0.58a	0.67±1.15b	-	5.02±2.48	49.32	-0.12	-1.00
		E2	6.67±0.58a	0.67±0.58b	-	4.36±2.56	58.68	-0.01	-0.66
	SPADS	E1	60.48±1.19a	51.30±0.66b	-	48.31±5.49	11.37	-0.13	0.26
		E2	60.51±0.82a	52.39±1.40b	-	55.53±7.17	12.91	-0.62	0.76
	SPADM	E1	41.33±10.68a	9.39±0.80b	-	22.42±10.43	46.50	0.34	0.33
		E2	42.64±2.55a	10.33±4.10b	-	20.05±13.05	65.12	0.93	-0.27
	GLAD(cm^2^)	E1	2979.43±389.52a	1153.23±135.83b	-	2817.07±897.31	31.85	0.46	0.46
		E2	3052.13±269.74a	1406.26±13.91b	-	3179.24±1269.91	39.94	0.55	-0.25
	GYP(g)	E1	84.83±7.31a	65.23±4.12b	-	53.73±26.60	49.52	0.22	-0.40
		E2	85.13±3.68a	64.31±4.42b		63.87±38.41	60.13	0.54	-0.23
TXF_2_	VSG(%)	E1	59.40±4.57a	-	18.98±1.42b	55.82±28.06	50.27	-0.25	-0.91
		E2	69.26±8.91a	-	21.48±1.28b	49.59±23.57	47.54	-0.73	-0.15
	GLNM	E1	6.33±0.58a	-	1.67±0.58b	5.23±2.86	54.75	-0.06	-0.84
		E2	6.67±0.58a	-	1.33±0.58b	5.14±2.45	47.58	-0.62	-0.10
	SPADS	E1	60.48±1.19a	-	41.48±3.37b	45.01±7.89	17.53	0.07	-0.59
		E2	60.51±0.82a	-	42.86±3.14b	49.67±6.93	13.96	-0.25	-0.01
	SPADM	E1	41.33±10.68a	-	12.92±4.21b	19.54±12.67	64.84	0.57	-0.98
		E2	42.64±2.55a	-	10.70±0.85b	14.40±13.08	90.82	0.99	-0.31
	GLAD(cm^2^)	E1	2979.43±389.52a	-	1633.99±213.56b	2767.59±726.59	26.25	0.33	0.52
		E2	3052.13±269.74a	-	1489.94±297.46b	4160.24±1369.63	32.92	0.35	0.19
	GYP(g)	E1	84.83±7.31a	-	48.40±0.66b	73.52±24.15	32.85	0.55	-0.05
		E2	85.13±3.68a	-	46.94±0.69b	57.08±30.26	53.01	0.11	-0.84

Note: E1: 2021, Changsha; E2: 2022, Changsha. Different letters following values within same row indicate significant differences between parents (*P*<0.05).

**Fig 1:**
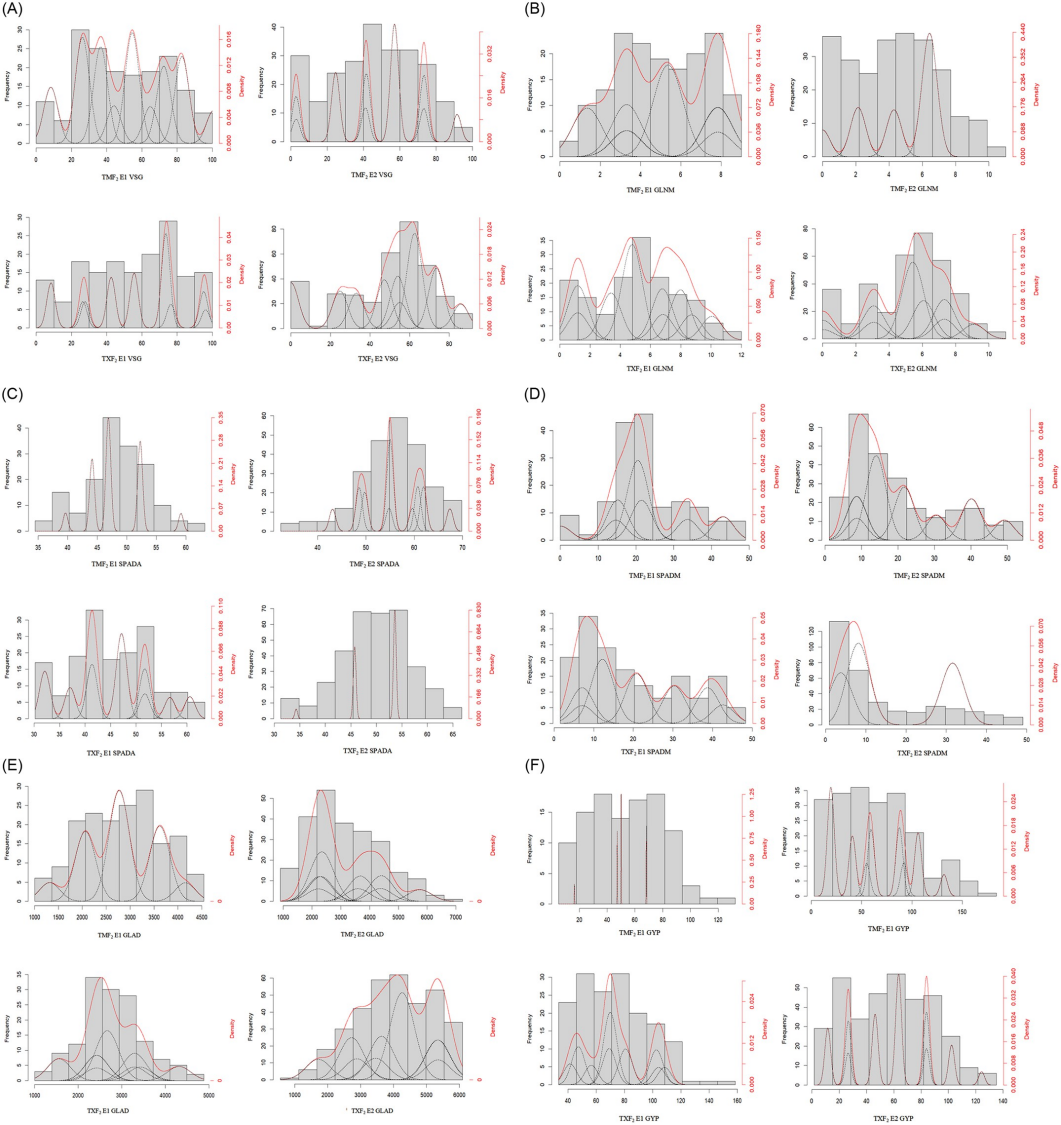
Frequent (column)and densed (red line) distributions for stay-green-related traits and grain yield per plant in TMF_2_ and TXF_2_ maize population. The white circle represents T01, the black solid circle represents Mo17 and Xin3, respectively.

The analysis of variance revealed that the VSG, GLNM, SPADS, SPADM, GLAD, and GYP of T01 in the TXF_2_ population were significantly higher than Xin3 under the two environments. There are varying degrees of bidirectional transgressive segregation phenomena in the six traits of TXF_2_ population. Their CV ranged from 13.96 to 90.82% (Table 1) in descending order from SPADM, VSG, GLNM, GYP, GALD, SPADS, to SPADM. The distribution of the six traits displayed a continuous variation with continuous bimodal or multimodal characteristics (Fig 1).

### Correlation analysis between the stay-green-related traits and grain yield per plant

**Fig 2:**
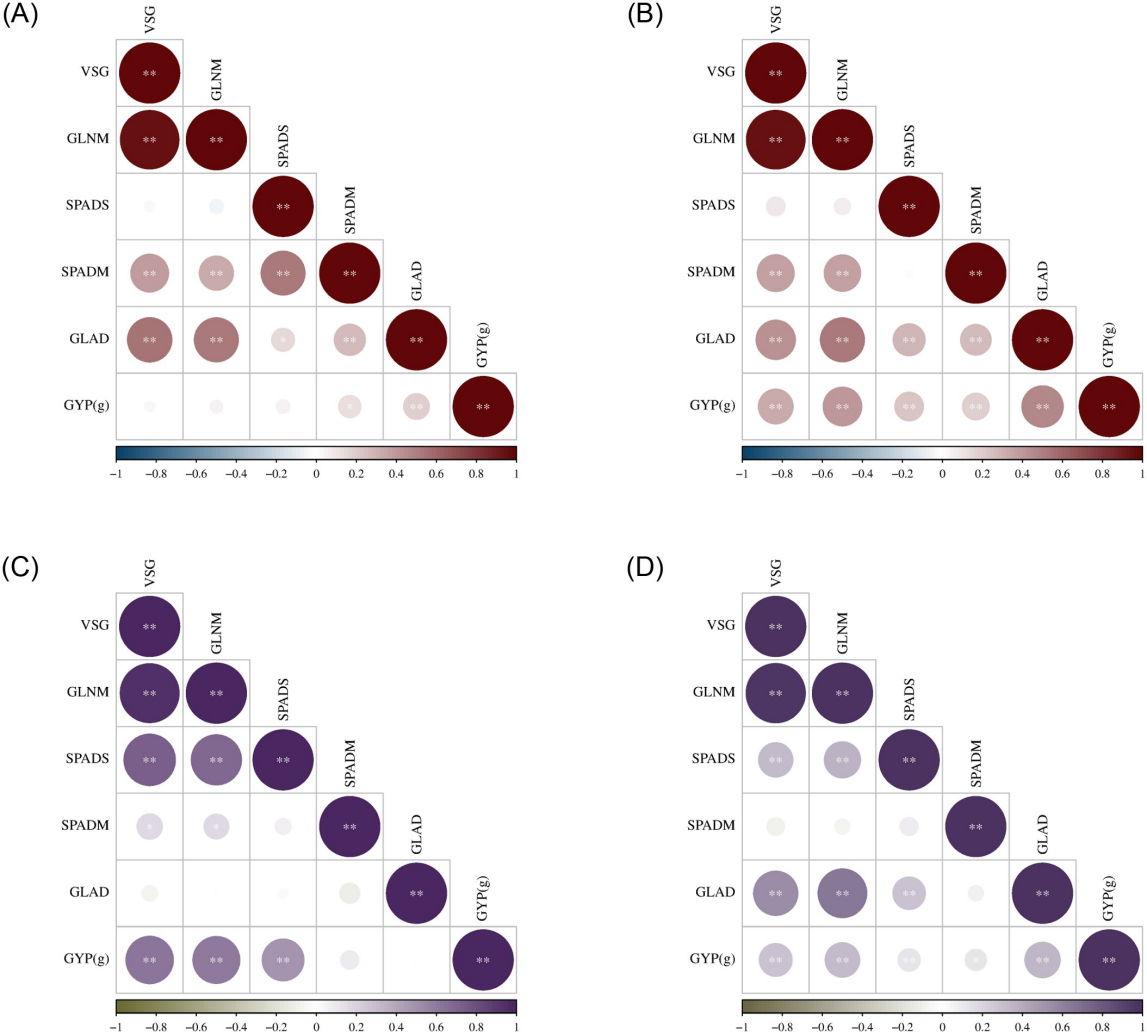
Correlation analysis of stay green related traits and grain yield per plant in TMF_2_ and TXF_2_ maize population. A, B: Correlation analysis of stay green related traits and grain yield per plant of TMF_2_ in two environments; C, D: Correlation analysis of stay green related traits and grain yield per plant of TXF_2_ in in two environments.

The correlations between VSG, GLNM, SPADS, SPADM, GLAD, and GYP are listed in Fig 2 (the correlation coefficient is shown in the S2 Table). The VSG in the TMF_2_ population was significantly positively correlated with GLNM, SPADM and GLAD, while GLNM and GLAD were significantly positively correlated under both environments. In the TXF_2_ population, VSG was significantly positively correlated with GLNM and GYP, GLNM with SPADS and GYP, and SPADS for GYP under both environments.

### The major gene and polygene mixed inheritance model for traits

Using SEA analysis software, major gene and polygene models were used to analyze the various traits of two populations, and 24 genetic models were obtained for each phenotype trait (S1 Table). Models with the lowest AIC value were selected as the candidate models (Table 2). In TMF_2_, models MX2-A-AD and MX2-ADI-AD had the lowest AIC values, with 1655.52 and 2375.58, respectively; hence, they were selected as the candidate models of VSG. For GLNM, the AIC values of MX2-ADI-AD and 2MG-CD models were the lowest (783.86 and 951.05, respectively) under the two environments, thus, they were selected as the candidate models of GLNM. In addition, 2MG-EA and 2MG-A with the lowest AIC values of SPADS (1045.61 and 1093.51, respectively) under the two environments were selected as the candidate models. The alternative SPADM models were MX2-ADI-AD, with AIC values of 1280.07 and 1915.85 in two environments, respectively. The lowest AIC value of GLAD in E1 was 3075.17, and the corresponding alternative model was 2MG-EA. However, the lowest AIC value of GLAD in E2 was 4323.81, and the alternative model was MX2-AD-AD. GYP alternative models were 2MG-EA and 2MG-A, with AIC values of 1054.14 and 2180.35 in E1 and E2, respectively.

**Table 2:** AIC values for segregation analysis of the optimal genetic models for stay-green-related traits and yield per plant in TMF_2_ and TXF_2_ maize population.

Population	Model	Environment	AIC					
			VSG	GLNM	SPADS	SPADM	GLAD	GYP
TMF_2_	1MG-AD	E1	1668.66	907.68	1054.75	1293.82	3084.83	1056.31
		E2	2585.78	1374.18	1700.24	1916.89	4325.39	2186.87
	1MG-EAD	E1	1672.26	1003.74	1060.05	1305.01	3085.68	1054.76
		E2	2776.03	1575.63	1701.21	1951.74	4325.75	2194.57
	2MG-A	E1	1682.92	787.23	1048.03	1420.17	3078.65	3701.93
		E2	2404.24	1227.10	1693.51*	1941.16	4325.41	2180.35*
	2MG-EA	E1	1668.57	788.81	1045.61*	1302.14	3075.17*	1054.14*
		E2	2422.36	1232.66	1701.80	1936.77	4323.83	2218.07
	2MG-CD	E1	1672.73	833.16	1102.21	1336.13	3103.49	1138.33
		E2	2410.64	951.05*	1744.42	1978.32	4340.47	2264.60
	2MG-EAD	E1	1661.61	807.95	1046.85	1280.83	3085.94	1054.55
		E2	2415.91	1169.69	1697.22	1962.54	4332.28	2191.11
	MX2-ADI-ADI	E1	1683.62	790.34	1060.26	1283.89	3088.98	1078.71
		E2	2382.67	1160.27	1715.00	1923.03	4337.82	2201.18
	MX2-ADI-AD	E1	1665.81	783.86*	1053.65	1280.07*	3084.06	1072.89
		E2	2375.58*	1155.19	1709.02	1915.85*	4332.39	2187.50
	MX2-AD-AD	E1	1656.62	838.70	1062.77	1398.03	3094.55	1069.99
		E2	2416.98	1176.44	1703.55	2018.04	4323.81*	2280.37
	MX2-A-AD	E1	1655.52*	840.81	1054.89	1399.21	3097.58	1158.44
		E2	2376.02	1158.18	1721.06	2087.53	4344.92	2283.52
TXF_2_	1MG-AD	E1	1630.88	952.12	1188.81	1284.27	2616.79	1568.13
		E2	3501.12	1625.88	2394.57	2563.34*	6334.78	3414.09
	1MG-EAD	E1	1632.27	1067.70	1187.83	1297.04	2789.55	1568.36
		E2	3806.21	1624.87	2393.54	2594.49	6593.98	3412.66
	2MG-A	E1	1630.64	846.16	1181.73	1292.13	2549.85	1565.82*
		E2	3196.86	1650.42	2403.13	2637.95	6129.94	3415.51
	2MG-EA	E1	1642.51	850.01	1189.01	1329.86	2549.01	1577.99
		E2	3275.74	1631.75	2387.57	2848.95	6146.19	3456.39
	2MG-CD	E1	1668.43	856.24	1259.63	1352.84	2567.59	1598.74
		E2	3287.20	1644.09	2437.34	2673.77	6153.14	3497.64
	2MG-EAD	E1	1624.98	868.37	1180.62	1335.74	2552.39	1575.28
		E2	3248.40	1592.71	2382.23*	2763.56	6135.32	3418.71
	MX2-ADI-ADI	E1	1641.80	848.45	1189.17	1285.03	2554.79	1584.58
		E2	3173.17	1582.85*	2398.05	2578.31	6133.19	3429.19
	MX2-ADI-AD	E1	1624.13*	842.08*	1179.27*	1282.54*	2548.87*	1578.35
		E2	3163.94*	1587.62	2402.95	2572.12	6126.49*	3405.00*
	MX2-AD-AD	E1	1630.19	862.14	1199.56	1405.78	2567.77	1618.86
		E2	3296.59	1652.29	2457.78	2933.07	6140.93	3508.59
	MX2-A-AD	E1	1629.38	867.19	1255.83	1410.97	2571.07	1608.35
		E2	3355.72	1692.82	2458.29	2985.07	6145.52	3517.03

MG: Major gene model; MX: Mixed major gene and polygene model. A: Additive effect; I: Interaction; E: Equal; ED: Epistasis dominanc.

*: The relatively lowest AIC values.

According to the fitness testing, the homogeneity, Smirnov, and Kolmogorov tests were used to determine the best models. Select the candidate model with less significant levels. Fitness testing of the best models identified MX2-A-AD as the appropriate VSG model, which described inheritance by major additive gene plus additive, dominant polygene, while MX2-ADI-AD was selected as the appropriate model for GLNM (Table 3). Moreover, the number of significant statistics of candidate SPADS models in the TMF_2_ population under both environments was 0. Based on the AIC value after evaluation, the appropriate SPADS models were 2MG-EA. Similar, the appropriate SPADS models were MX2-ADI-AD models. At the same time, 2MG-EA, the model for two pairs of additive allele major gene inheritance, was selected as the appropriate model for GLAD and GYP.

**Table 3:** Adaptability test of the optimal genetic models for stay-green-related traits and yield per plant in TMF_2_ and TXF_2_ maize population.

Population	Traits	Environment	Model code	Generation	U_1_^2^	U_2_^2^	U_3_^2^	nW^2^	Dn
TMF_2_	VSG	E1	MX2-A-AD	P_1_	0.008	0.062	1.773	0.101	0.401
				P_2_	0.055	0.296	1.609	0.167	0.395
				F_1_	0.036	0.004	0.965	0.128	0.448
				F_2_	0.001	0.003	0.013	0.042	0.043
	GLNM	E1	MX2-ADI-AD	P_1_	0.066	0.003	0.617	0.106	0.340
				P_2_	0.000	0.059	0.942	0.080	0.245
				F_1_	0.000	0.390	6.250*	0.417	0.500
				F_2_	0.000	0.001	0.006	0.201	0.079
	SPADS	E1	2MG-EA	P_1_	0.074	0.189	0.469	0.175	0.377
				P_2_	0.044	0.076	0.083	0.050	0.207
				F_1_	0.082	0.017	0.348	0.167	0.388
				F_2_	0.600	0.452	0.097	0.115	0.071
	SPADM	E1	MX2-ADI-AD	P_1_	0.225	0.005	4.473*	0.192	0.366
				P_2_	0.043	0.023	0.038	0.071	0.233
				F_1_	0.003	0.098	1.051	0.049	0.229
				F_2_	0.000	0.001	0.003	0.027	0.039
		E2	MX2-ADI-AD	P_1_	0.001	0.039	0.888	0.038	0.212
				P_2_	0.029	0.059	0.098	0.072	0.290
				F_1_	0.001	0.005	0.036	0.029	0.201
				F_2_	0.001	0.000	0.012	0.021	0.024
	GLAD	E1	2MG-EA	P_1_	0.001	0.019	0.449	0.063	0.266
				P_2_	0.226	0.348	0.271	0.175	0.377
				F_1_	0.078	0.132	0.138	0.152	0.353
				F_2_	0.002	0.001	0.001	0.036	0.042
	GYP	E1	2MG-EA	P_1_	0.012	0.000	0.191	0.045	0.272
				P_2_	0.047	0.018	0.092	0.066	0.288
				F_1_	0.021	0.064	0.206	0.056	0.314
				F_2_	0.396	0.571	0.343	0.077	0.071
TXF_2_	VSG	E1	MX2-ADI-AD	P_1_	0.109	0.114	0.006	0.097	0.331
				P_2_	0.035	0.001	0.578	0.086	0.244
				F_1_	0.001	0.009	0.2533	0.078	0.244
				F_2_	0.001	0.004	0.016	0.037	0.051
	GLNM	E1	MX2-ADI-AD	P_1_	0.002	0.448	6.322*	0.157	0.338
				P_2_	0.000	0.387	6.250*	0.417	0.500
				F_1_	0.000	0.384	6.250*	0.417	0.501
				F_2_	0.000	0.002	0.000	0.147	0.071
	SPADS	E2	2MG-EAD	P_1_	0.014	0.056	0.235	0.037	0.168
				P_2_	0.001	0.025	0.287	0.051	0.220
				F_1_	0.060	0.058	0.000	0.062	0.251
				F_2_	0.000	0.003	0.045	0.030	0.026
	SPADM	E1	MX2-ADI-AD	P_1_	0.002	0.040	0.931	0.050	0.242
				P_2_	0.177	0.306	0.342	0.153	0.362
				F_1_	0.102	1.015	7.829*	0.218	0.393
				F_2_	0.008	0.002	0.027	0.025	0.038
	GLAD	E1	MX2-ADI-AD	P_1_	0.012	0.250	5.903	0.163	0.315
				P_2_	0.003	0.116	1.359	0.105	0.296
				F_1_	0.072	0.173	7.321*	0.506*	0.532
				F_2_	0.004	0.007	0.007	0.018	0.030
	GYP	E1	2MG-A	P_1_	0.056	0.099	0.114	0.079	0.296
				P_2_	0.000	0.406	6.495*	0.278	0.381
				F_1_	0.006	0.078	0.672	0.051	0.259
				F_2_	0.003	0.001	0.007	0.009	0.023

U_1_^2^, U_2_^2^, U_3_^2^: The statistic of Uniformity test; nW^2^: The statistic of Smirnov test; Dn: The statistic of Kolmogorov test;

*: Significant difference at 0.05 probability level.

Furthermore, the AIC value (Table 2) and fitness test (Table 3) analyses revealed that the optimal inheritance models for VSG, GLNM, SPADM and GLAD in the TXF_2_ population were MX2-ADI-AD, implying that the inheritance of these four traits was controlled by additive-dominance-epistasis major gene plus additive-dominance polygene genetic. However, the optimal inheritance model for SPADS was 2MG-EAD, implying that two pairs of additive-dominant allele genes controlled the SPADS inheritance. The optimal model for GYP was 2MG-A, regulated by two pairs of additive major gene inheritance.

### Estimation of genetic parameters

The first-order and second-order parameters for traits estimated from their optimal inheritance models are listed in Tables 4 and 5. For the SPADS, GLAD and GYP additive effects in the TMF_2_ population, there was a |*da*| = |*db*| relationship, implying equal additive effects of the first and second major genes. Moreover, the additive effects of the first major gene were larger than the second major gene for VSG, GLNM and SPADM in E1. For the dominant effects (*ha* and *hb*) on SPADM in TMF_2_, the first major gene contributed more than the second major gene. However, the second major gene generated more dominant effects on GLNM characteristics than the first. For the epistatic effects, the additive × additive (*i*), dominance× dominance (*l*), additive× dominance (*jab*) and dominance × additive (*jba*) interactions between the two major genes were evident. SPADM had the most significant additive× additive effects (*i* = 16.74) among all the tested traits. Similarly, SPADM had the largest inhibitive dominance × dominance effect (*l* = 21.53) compared to the other traits. The major-gene heritability of the six tested traits was greater than the polygene heritability, implying that these six traits were mainly controlled by major genes and slightly modified by polygenes.

**Table 4:** The first-order parameters of the best model for stay-green-related traits and yield per plant in TMF_2_ and TXF_2_ maize population.

Population	Traits	Environment	Model code	First order parameters									
				*da(d)*	*db*	*ha(h)*	*hb*	*i*	*jab*	*jba*	*l*	*[d]*	*[h]*
TMF_2_	VSG	E1	MX2-A-AD	28.28	17.95	-	-	-	-	-	-	-18.34	42.74
	GLNM	E1	MX2-ADI-AD	4.62	2.36	-2.36	-3.34	2.36	-1.38	-0.10	3.13	-4.31	7.26
	SPADS	E1	2MG-EA	3.04	-	-	-	-	-	-	-	-	-
	SPADM	E1	MX2-ADI-AD	16.74	7.25	-22.59	-19.35	16.74	4.79	-4.07	21.53	-6.83	63.27
	GLAD	E1	2MG-EA	876.17	-	-	-	-	-	-	-	-	-
	GYP	E1	2MG-EA	3.17	-	-	-	-	-	-	-	-	-
TXF_2_	VSG	E1	MX2-ADI-AD	29.65	4.62	-7.61	-4.95	5.24	13.73	1.90	29.46	-6.06	20.30
	GLNM	E1	MX2-ADI-AD	1.74	-1.07	-1.61	-2.11	2.69	1.65	2.77	1.81	2.50	6.45
	SPADS	E2	2MG-EAD	3.62	-	-	-	-	-	-	-	-	-
	SPADM	E1	MX2-ADI-AD	-5.16	-16.78	-27.67	-18.72	22.94	20.99	23.66	16.94	37.86	73.71
	GLAD	E1	MX2-ADI-AD	440.14	5.00	-938.71	-935.77	505.68	425.93	-2.98	1176.73	1178.49	2109.62
	GYP	E1	2MG-A	26.56	-6.84	-	-	-	-	-	-	-	-

*da*: Additive effect of the first major gene; *db*: Additive effect of the second major gene

**Table 5:** The second-order parameters of the best model for stay-green-related traits and yield per plant in TMF_2_ and TXF_2_ maize population.

Population	Traits	Environment	Model code	Second order parameters			
				*σ 2 mg*	*h 2 mg (%)*	*σ 2 hg*	*h 2 pg (%)*
TMF_2_	VSG	E1	MX2-A-AD	631.67	95.95	10.32	1.57
	GLNM	E1	MX2-ADI-AD	5.32	89.03	0.11	1.78
	SPADS	E1	2MG-EA	29.99	99.43	-	-
	SPADM	E1	MX2-ADI-AD	100.8	92.78	0.00	0.00
	GLAD	E1	2MG-EA	652962.40	93.47	-	-
	GYP	E1	2MG-EA	707.77	99.99	-	-
TXF_2_	VSG	E1	MX2-ADI-AD	748.54	95.08	32.92	4.18
	GLNM	E1	MX2-ADI-AD	7.40	90.16	0.35	4.26
	SPADS	E2	2MG-EAD	47.85	99.55	-	-
	SPADM	E1	MX2-ADI-AD	152.44	94.92	-	-
	GLAD	E1	MX2-ADI-AD	447104.80	84.46	11312.13	2.14
	GYP	E1	2MG-A	560.88	96.17	-	-

*σ*^*2*^ *mg*: Major gene variance; *h* ^*2*^ *mg (%)*: Heritability of major gene; *σ*^*2*^ *hg*: polygene variance; *h* ^*2*^ *pg (%)*: Heritability of polygene-Var;“-”in the cells mean the value is absent.

The MX2-ADI-AD model regulated the heredity of VSG in the TXF_2_ population. In addition, the additive effects of the first and second major genes were positive (*da* = 29.65, *db* = 4.62). However, the additive genetic contribution rate of the first major gene was greater than the second major gene (|*da*| > |*db*|). At the same time, the additive effects of the two major genes were higher than the dominant effects (|*da*|+|*db*| > |*ha*|+|*hb*|), implying that the inheritance of VSG was mainly due to a positive additive effect. Moreover, the epistatic effect values of additive×additive (*i* = 5.24), dominance×dominance (*l* = 29.46), additive×dominant (*jab* = 13.73) and dominant×additive were positive (*jba* = 1.9), with 95.08% heritability of the major gene. Similarly, the inheritance of GLNM was also modeled with MX2-ADI-AD, with da, and db values of 2.73 and 0.57, respectively. The heritability of the major gene was 90.16%. In addition, the heredity of SPADS was controlled by two pairs of additive-dominant allelic genes, with a 99.55% heritability of the major genes. In contrast, the heritability of the SPADM major gene was 94.92%, with the same additive effect values of the major genes as GLNM, mainly a negative genetic effect. The inheritance of GLAD was fitted to a model for two pairs of the additive-dominance-epistasis major gene plus additive-dominance polygene genetic with *da* = 440.14. The heredity of GYP was controlled by two pairs of major genes, *da* = 25.56 and *db* = -5.84, respectively, in E1.

## Discussion

The stay-green-related trait is an important factor influencing the maize yield. The most significant change in plant morphology during the improvement process of maize varieties is the enhanced stay green leaves in the later stage of grain filling. This, in turn, extends the effective photosynthetic period of leaves and increases dry matter accumulation by 2.13 times [26]. Lee et al. [27] argues that the stay green traits has played a crucial role in the significant increase in yield of hybrid maize varieties in the United States from 1939 to the present. Other studies have reported that the later the aging of maize leaves occurs, the slower their aging rate will be, this phenomenon is more conducive to enhancing grain weight and the number of grains per ear [28].Therefore, establishing the genetics of stay-green traits is significant in improving and stabilizing maize yield. Most studies on the stay green traits have focused on QTL mapping analysis [29–34]. A previous study by Zheng et al. [29] identified 14 QTLs for green leaf area in F_2_ populations of Q319 and Mo17 maize inbred lines. In addition, Wang et al. [35] revealed that three main QTLs control the stay green leaf area, while Belicuas et al. [36] identified 17 green QTLs through composite interval mapping, most of which were concentrated on chromosomes 1, 2, 3 and 4. Chang et al. [37] also used QTL mapping to parse the genetic variance of visual stay green, green leaf number at the silking stage and green leaf number at the mature stage of maize, a total of 37 QTLs were detected on all chromosomes except Chromosome 10, with a 4.34~22.40% phenotypic variation contribution rate. However, few studies have explored the inheritance of stay-green-related traits using the major mixed gene plus polygene inheritance model. The major gene plus polygene model is widely used to analyze the genetic composition of quantitative crop traits. It could preliminarily reveal the genetic basis of the target traits using phenotype data while verifying the results from QTL mapping, providing a basis for QTL mapping of quantitative traits.

This study analyzed the phenotypic data of six traits from two populations under two environments using the mixed inheritance model of major gene plus polygene. Under the different environments, the impact of the environment is avoided while the two segregating populations, TMF_2_ and TXF_2_ simultaneously analyzed, increasing the accuracy of the results. Besides, the different stay green related traits determined the stay green genetic effect, improving the reliability of the mixed inheritance model of the major gene plus polygene results. However, this study was done in a single location using only the F_2_ hybrid. In future research, a longer experimentation period, more locations, and F_2:3_ families should be explored to increase the statistical power and further clarify the inheritance of the stay-green-related traits. Further experiments with extensive research materials will deepen our understanding of the inheritance mechanism of the stay-green trait.

The correlation between stay green traits and yield is generally consistent in both environments. 2 populations concluded that yield is positively correlated with VSG, GLNM, SPADS, SPADM, and GLAD. In addition, these traits had the same best inheritance model. Therefore, the breeding process should fully consider the correlation between these traits to improve the selection efficiency of target traits.

Previous studies have used single identification indicators, such as the number of green leaves at maturity, the duration of absolute green leaf area, the relative green leaf area at maturity, and the chlorophyll content to evaluate the green retention of various crops. However, there are few studies on the gene effect and genetic efficacy of maize green retention using the above multiple indicators. Moreover, there are many studies on the genetic characteristics of maize chlorophyll content and the mechanism of gene action. For example, Bao et al. [38] revealed that the leaf chlorophyll content model of six generations of hybrid popcorn combinations was controlled by a pair of additive-dominant major genes + additive-dominant epistatic polygenes, and the main gene heritability was greater than the polygene inheritance rate. Irfan et al. [39] also revealed that two gene pairs control the inheritance of chlorophyll content at the flowering stage. This implies that there is a major gene controlling the chlorophyll content traits. However, some studies have revealed that the genetic effect of chlorophyll is mainly additive, and others are non-additive, which may be caused by a different selection of test materials, sampling period and analysis methods [40–43]. In addition, the VSG, GLNM and SPADM of the two populations are controlled by two major dominant genes + dominant polygenes, consistent with the previous research results. The results from this study provide important genetic information for breeding and lay a foundation for QTL mapping of stay green traits.

## Conclusions

This study synthesized a four-generation genetic analysis of two populations for stay green straits, indicating that stay green traits are typical quantitative genetic traits, in maize two F_2_ populations, ideal models and genetic influences for stay-green traits were found. The two main genes of the additive effect additive dominant polygenic model control the optimal genetic models for VSG, GLNM, and SPADM in the two populations. High heredity and little environmental influence are both characteristics. Two major genes of equal-additive dominance effects controlled GLAD in TMF_2_. In TXF_2_, GLAD was regulated by two major genes of additive-dominance interaction effects plus additive-dominance polygene model. The results of this study lay a solid foundation for identifying key genes and developing associated molecular markers for the stay green trait in maize.

## Supporting information

S1 Table Analysis of genetic model of traits. (XLSX)

S2 Table Correlation coefficient of stay green related traits and grain yield per plant in TMF_2_ and TXF_2_ maize population. (XLSX)
